# Prediction of reoffending risk in men convicted of sexual offences: development and validation of novel and scalable risk assessment tools (OxRIS)

**DOI:** 10.1016/j.jcrimjus.2022.101935

**Published:** 2022

**Authors:** Rongqin Yu, Yasmina Molero, Niklas Långström, Thomas Fanshawe, Denis Yukhnenko, Paul Lichtenstein, Henrik Larsson, Seena Fazel

**Affiliations:** a Department of Psychiatry, University of Oxford, Oxford, UK; b Department of Clinical Neuroscience, Karolinska Institutet, Stockholm, Sweden; c Department of Medical Epidemiology and Biostatistics, Karolinska Institutet, Stockholm, Sweden; d National Board of Health and Welfare, Stockholm, Sweden; e Nuffield Department of Primary Care Health Sciences, University of Oxford, UK

**Keywords:** Risk assessment, Clinical algorithms, Big data, Sexual offending, Prediction model

## Abstract

**Background:**

Current risk assessment tools have a limited evidence base with few validations, poor reporting of outcomes, and rarely include modifiable factors.

**Methods:**

We examined a national cohort of men convicted of sexual crimes in Sweden. We developed prediction models for three outcomes: violent (including sexual), any, and sexual reoffending. We used Cox proportional hazard regression to develop multivariable prediction models and validated these in an external sample. We reported discrimination and calibration statistics at prespecified cut-offs.

**Findings:**

We identified 16,231 men convicted of sexual offences, of whom 14.8% violently reoffended during a mean follow up of 38 months, 31.4% for any crime (34 months), and 3.6% for sexual crimes (42 months). Models for violent and any reoffending showed good discrimination and calibration. At 1, 3, and 5 years, the area under the curve (AUC) was 0.75–0.76 for violent reoffending and 0.74–0.75 for any reoffending. The prediction model for sexual reoffending showed modest discrimination (AUC = 0.67) and good calibration. We have generated three simple and web-based risk calculators, which are freely available.

**Interpretation:**

Scalable evidence-based risk assessment tools for sexual offenders in the criminal justice system and forensic mental health could assist decision-making and treatment allocation by identifying those at higher risk, and screening out low risk persons.

## Highlights

• Prediction models for violent and any reoffending in sex offenders were developed.
• The new tools demonstrated good calibration, particularly at lower risk levels.
• Discrimination was good for violent and any reoffending.
• These models were translated into freely available online risk calculators.

## 1 Introduction

Reducing repeat offending in individuals convicted of sexual offences is part of targeted strategies to reduce sexual violence, and a major focus for the criminal justice systems and public health (Matravers, 2013; McCartan & Laws, 2018). Repeat offending, also known as recidivism, remains high in most countries with rates typically of 40–60% within two years (Yukhnenko, Sridhar, & Fazel, 2019). Managing risk partly relies on accurate risk assessment (Zavrsnik, 2021), which need to be linked to effective interventions. Accurately differentiating people convicted of sexual crimes for reoffending risk could also potentially inform sentencing, needs assessment, and resource allocation.

Many instruments are available for assessing reoffending risk in individuals convicted of sexual crimes, and have been found to be more accurate than unstructured clinical judgement (Hanson & Kelly, 2009). Although widely used, the most common tools (Hanson, 1997; Hanson & Thornton, 1999; Harris, Rice, & Quinsey, 1993; Quinsey, Harris, Rice, & Cormier, 1998) were developed more than two decades ago and based on small samples. External validations, particularly when tool developers were not involved, have reported low to moderate predictive accuracy (Azizian, Olver, Rokop, & D'Orazio, 2021; Boccaccini, 2017; Hanson & Morton-Bourgon, 2009; Helmus, Hanson, Murrie, & Zabarauckas, 2021; Reeves, Ogloff, & Simmons, 2018; Tully, Chou, & Browne, 2013). Almost all studies have not reported key performance measures such as calibration, and true and false positives rates. In addition, current tools often require extensive training to administer, are paper-and-pencil based, and involve lengthy interviews of perpetrators or victims (Hanson & Morton-Bourgon, 2009; Tully et al., 2013). Some tools also rely on scores from other scales, such as the Sex Offender Risk Appraisal Guide (SORAG; Quinsey et al., 1998) that includes a Psychopathy Checklist score (Hare, 2003) making it resource intensive. Furthermore, actuarial tools classify people into broad risk categories, such as high and low, and do not include probability scores. As there are different understandings of what these categories mean in practice, this approach is liable to bias and results in poor consistency of risk predictions (Thornton, 2017). Further, most commonly used tools only include sociodemographic and criminological factors (Hanson & Thornton, 1999), providing limited assistance with treatment planning, where the inclusion of modifiable factors can assist.

This study aims to develop risk prediction models for reoffending outcomes in individuals convicted of sexual crimes. We linked data from a large nationwide population-based cohort of all people convicted of sexual offences over a period of 20 years to examine a wide range of empirically supported or theoretically based reoffending risk factors (Hanson & Morton-Bourgon, 2009; Lee & Hanson, 2016). These risk factors include sociodemographic characteristics such as age, education, marital status (Andersen, Andersen, & Skov, 2015), and employment status, criminological factors such as index crime category and history, and mental health problems including alcohol and drug use disorders. Their association with criminal recidivism outcomes have been empirically tested with evidence summarized in systematic reviews (Fazel & Yu, 2011; Piquero, Jennings, Diamond, & Reingle, 2015; Yu, Geddes, & Fazel, 2012).

## 2 Methods

From the National Crime Register, we identified a national cohort of all men (from 15 years old, i.e. the age of criminal responsibility in Sweden) convicted of sexual offences in Sweden between 1994 and 2013; the 20-year follow up started from 1 January 1994 and ended on 31 December 2013. Sexual offending included all crimes that were defined as a sexual offence according to Swedish law, such as rape, sexual coercion, child sexual abuse, and sexual harassment (See Appendix Table 1 for a complete list). It was not possible to calculate the frequency for certain individual categories due to overlapping crime codes although data for buying of a sexual service from an adult was available (*n* = 1554 [9.6%] out of 16,231 men). We followed up each man from the conviction date of one randomly selected sexual crime from 1994 to 2013 to first future reoffending date (crime date rather than conviction date), death, or to end of the study (31 December 2013). To assure selection of a representative sample of men convicted of sexual crime in the registry, we chose a randomly selected sexual crime, rather than the first conviction as the index conviction, in order to avoid overrepresentation of men convicted of sexual crime for the first time in the cohort and provide for a more representative sample. We obtained information on crimes from the National Crime Register, which includes all convictions in Swedish district courts. The crime register has total national coverage – only 0.05% of all registered convictions had incomplete personal identification numbers (Fazel & Grann, 2006). The study was approved by the Regional Ethics Committee at Karolinska Institutet, Sweden (2013/5:8).

#### 2.1 Measurement of risk factors

By using the unique personal identification number carried by all citizens of Sweden, we linked data from high-quality national prospective registers with mandatory reporting. We tested a range of sociodemographic, criminological, and mental health status-related risk factors suggested in prior empirical and theoretical work for possible inclusion in prediction models.

Sociodemographic factors were age, single status, education level, and unemployment when convicted of sexual offence (see Appendix for definitions and Table 1 for basic characteristics). Data were collected from the Longitudinal Integrated Database for Health Insurance and Labour Market Studies (LISA) register (Ludvigsson, Svedberg, Olen, Bruze, & Neovius, 2019). Criminological factors included prior convictions of sexual offence before the index sexual offence, previous non-sexual violence, and previous non-violent offending, and previous imprisonment. Mental health variables included any alcohol use disorder, drug use disorder, and a category of any other mental disorders. We also examined previous violent victimization and parental violence conviction (through linkage with the Multi-Generation Register at Statistics Sweden; Ekbom, 2011) as risk factors.

**Table 1:** Baseline characteristics of individuals convicted of sexual offences and risk factors

	Derivation sample	Validation sample
	*n* = 12,674	*n* = 3557
Age	37.7 (15–91)^a^	38.2 (15–90)
Unemployment^b^		
No	5551 (45.4%)	1577 (45.9%)
Yes	6678 (54.6%)	1862 (54.1%)
Single status^b^		
No	2701 (22.1%)	812 (23.6%)
Yes	9528 (77.9%)	2627 (76.4%)
Education		
Secondary	5206 (49.4%)	1515 (49.7%)
Upper-secondary	4548 (43.1%)	1342 (44.0%)
Post-secondary	789 (7.5%)	192 (6.3%)
Previous sexual offence	766 (6.0%)	218 (6.1%)
Previous nonsexual violence	3149 (24.9%)	841 (23.6%)
Previous nonviolent offence	6916 (54.6%)	1912 (53.8%)
Prior imprisonment	893 (7.1%)	242 (6.8%)
Alcohol use disorder^c^	1505 (11.9%)	380 (10.7%)
Drug use disorder^c^	891 (7.0%)	220 (6.2%)
Any mental health disorders^d^	1896 (15.0%)	574 (16.1%)
Previous violent victimization	699 (5.5%)	170 (4.8%)
Parental violence conviction	1149 (9.1%)	340 (9.6%)

Note: ^a^ Mean (range); ^b^ status at time of conviction of sexual offence; ^c^ Refers to ICD clinical diagnosis before conviction of sexual offence; ^d^ Any mental disorders other than alcohol use disorders and drug use disorders.

We identified lifetime diagnoses of psychiatric disorders before conviction of sexual offence using the International Classification of Diseases (ICD) codes recorded in the Swedish National Patient Register (Ludvigsson et al., 2011). The Swedish National Patient Registers provide ICD diagnoses for all inpatient psychiatric hospital admissions in Sweden since 1973 and specialist outpatient care since 2001. The registers adopted the 8th Revision (ICD-8) between 1973 and 1986, the 9th Revision (ICD-9) between 1987 and 1996, and 10th Revision (ICD-10) since 1997. We investigated the following specific or groups of psychiatric disorders: (1) alcohol use disorder (ICD-8: 291, 303; ICD-9: 291, 303, 305A; ICD-10: F10); (2) drug use disorder (ICD-8: 304; ICD-9: 292, 304, 305 [except. A]; ICD-10: F11–F19); and (3) any mental disorder (including all applicable codes in ICD-8: 290–315; ICD-9: 290–319; ICD-10: F00–F99, excluding alcohol and drug use disorders). Previous violent victimization (ICD-9 codes: E960–E969; ICD-10 codes: X85–Y09) was identified as being hospitalized as a victim of violence, which will capture only severe cases, has high specificity but will underestimate victimization events.

Parental violence was defined as any violent conviction of either parent (identified through the Swedish Multi-Generation Register; Ekbom, 2011) before the index sex offence of the person investigated. This factor was tested based on previous work showing familial clustering of sexual offending, primarily accounted for by genes rather than shared environmental influences (Langstrom, Babchishin, Fazel, Lichtenstein, & Frisell, 2015).

#### 2.2 Measurement of outcomes

Primary outcomes were first violent (including sexual) reoffending after the index sexual crime at 1, 3, and 5 years, so that findings could be useful for monitoring both short- and longer-term outcomes. We used this as the primary outcome as sexual reoffending is often under-reported and classified as general violent reoffending in crime records (Rice, Harris, Lang, & Cormier, 2006). We examined both short and longer term recidivism outcomes to meet the needs of different services and agencies. Violent reoffending was defined as a conviction of any violent crime occurring after the index sexual offence conviction. Violent crimes included homicide, assault, robbery, arson, any sexual offence (rape, sexual coercion, child molestation, indecent exposure, or sexual harassment), illegal threats, or intimidation. During the follow up, to avoid pseudo-recidivism (where historical offences are convicted after the conviction of the more recent index offence), we adopted the actual crime date as the event date instead of the reconviction date. However, if the date of the reoffending was not recorded, reconviction date was used. We also included any reoffending (including violent and non-violent crimes), and sexual reoffending (rape, sexual coercion, child molestation, indecent exposure, or sexual harassment), as secondary outcomes.

#### 2.3 Statistical analyses

To examine the association between risk factors and reoffending outcomes and to account for time to event (reoffending crime date), we conducted multivariable Cox proportional hazard regression. We separated the samples into derivation and validation samples. The validation sample was a subsample selected using stratified random selection approach based on the residential regions (urban areas of major cities, suburbs of major cities, counties with medium populations, counties with small populations) of the individual at the year of conviction of sexual offence. This method was chosen over a random proportion of the overall sample in order to achieve a balance of urban high and rural low population areas, to test validation of the model in a population that was geographically distinct from the one in the derivation sample.

We used the derivation sample to generate models for predicting reoffending outcomes after conviction of the index sexual offence. We used the validation sample to test the performance of models from the derivation sample in predicting reoffending outcomes in an external sample.

Multiple imputation was used to calculate missing values for predictors, using regression models that used data from other risk factors and the outcome, with the Nelson-Aalen cumulative hazard function (White & Royston, 2009). We conducted 20 imputations and calculated coefficients by combining information across all imputed datasets. The final model included all selected variables that retained significance (*p* < .05) in multivariable analyses (see details of selection process in the Appendix Protocol). To examine the predictive ability of the identified final model, we tested both discrimination and calibration. We used Harrell's c-index as an overall measure of discrimination, defined as the ability of model to differentiate between individuals with and without reoffending outcomes during the follow-up. The c-index ranges from 0.5 to 1.0, with 1.0 representing perfect discrimination (Pencina & D'Agostino, 2004). For outcomes within a certain time period (1, 3, and 5 years) after the index sexual offence conviction, we calculated the areas under the receiver operating characteristics curve (AUC). We estimated the absolute predicted probabilities based on the regression model coefficients and baseline survivor function within a certain time period. We reported sensitivity, specificity, positive and negative predictive value (NPV) and their confidence intervals (CIs) based on prespecified thresholds (5%, 10%, 15%, and 20%) for all outcomes. Unlike sensitivity and specificity, PPV and NPV are sensitive to changes in base rate. For any given test (i.e., sensitivity and specificity remain the same); as prevalence decreases, the PPV deceases because there will be more false positives for every true positive, and the NPV increases because there will be more true negatives for every false negative.

In addition, we examined calibration, how close predicted risks were to observed ones, by plotting these risks against each other. We also calculated Brier scores (Brier, 1950), which is the average quadratic difference between the predicted probability and the observed binary outcome. The Brier score ranges from 0 to 1, with lower scores indicating better calibration.

We used the subsample generated based on geographical regions to validate the final models for violent, any, and sexual reoffending. We used STATA (version 16) for all analyses. Finally, we used the models for predicting different outcomes to develop three online risk calculators that provide probability scores at 1, 3, and 5 years.

## 3 Results

We identified a cohort of 16,231 men convicted of sexual crimes between 1994 and 2013. In the overall cohort, during a mean follow-up of 38 months, 2403 (15%) were reconvicted of violent offending, 5101 (31%, 34 months follow-up) of any offending, and 588 (3.6%, 42 months) of sexual reoffending. The derivation sample consisted of 12,674 men convicted of sexual offences and the external validation sample of 3557 men convicted of sexual offences, with similar baseline characteristics (Table 1). In the derivation sample, 1902 (15%) were reconvicted of violent offending and 4022 (32%) were reconvicted of any offending. Similar rates were found for the validation sample, in which 501 (14%) were reconvicted of violent offending and 1079 (30%) any reoffending. For sexual reoffending, rates were comparable in the derivation and validation samples (3.7% and 3.4%, respectively). Observed probabilities for violent reoffending, any reoffending, and sexual reoffending within 1, 3, and 5 years, respectively, after a sexual offence conviction are presented in Table 2 and Appendix Fig. 1.

**Table 2:** Rates of repeat offending for violent, any, and sexual crimes

	Derivation sample	Validation sample
	(*n* = 12,674)	(*n* = 3557)
Violent reoffending	1902 (15.0%)	501 (14.1%)
Within 1 year	547 (4.3%)	147 (4.1%)
Within 3 years	1173 (9.3%)	297 (8.4%)
Within 5 years	1504 (11.9%)	380 (10.7%)
Any reoffending	4022 (31.7%)	1079 (30.3%)
Within 1 year	1452 (11.5%)	367 (10.3%)
Within 3 years	2709 (21.4%)	700 (19.7%)
Within 5 years	3296 (26.0%)	866 (24.4%)
Sexual reoffending	467 (3.7%)	121 (3.4%)
Within 1 year	127 (1.0%)	28 (0.8%)
Within 3 years	265 (2.1%)	74 (2.1%)
Within 5 years	352 (2.8%)	91 (2.6%)

**Fig. 1:**
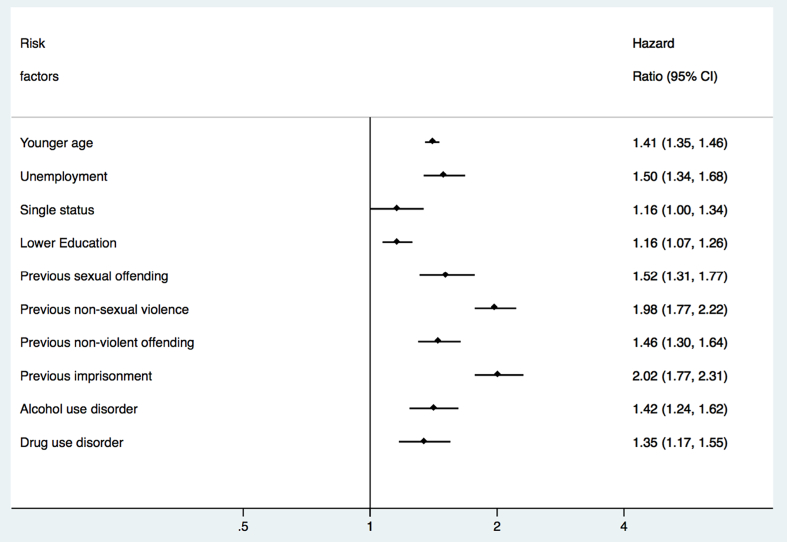
Risk factors included in the final model for prediction of violent reoffending and their hazard ratios. Note: Younger age refers to the effect per 10 years of age. Unemployment and single status refer to status at time of conviction of index sexual offence.

**Fig. 2:**
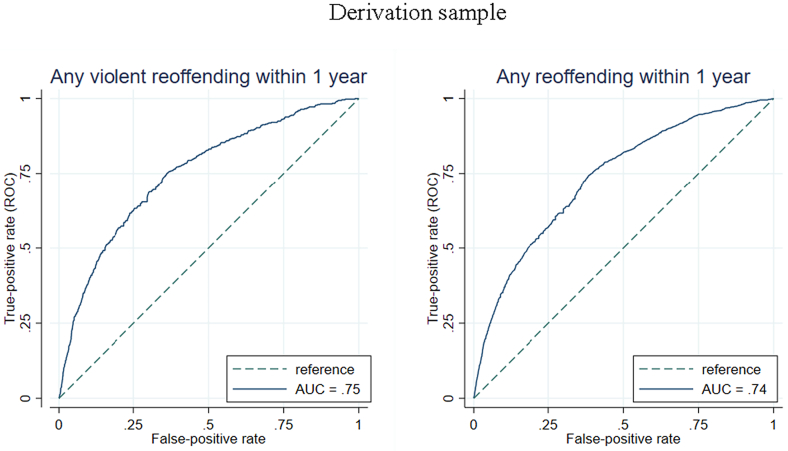
Model discrimination shown by receiver operating characteristics curves for violent and any reoffending within 1 year. Note: AUC = areas under the receiver operating characteristics curve.

**Fig. 3:**
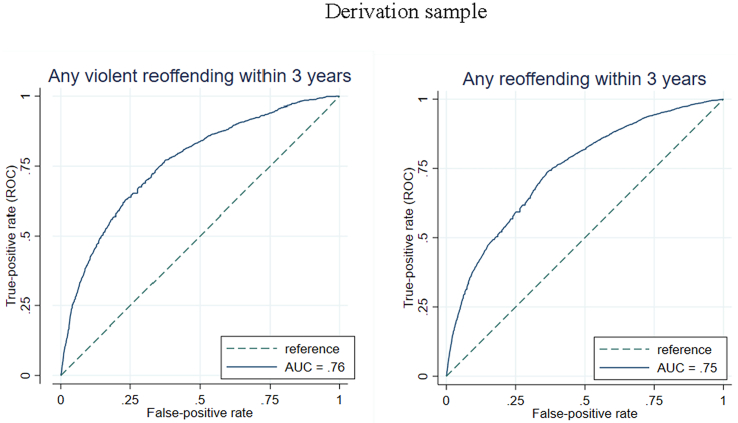
Model discrimination shown by receiver operating characteristics curves for violent and any reoffending within 3 years. Note: AUC = areas under the receiver operating characteristics curves.

The final model for violent reoffending included the following risk factors: younger age, unemployment, single status, lower education level, previous sexual offending, previous non-violent offending, previous imprisonment, a diagnosis of alcohol use disorder, and a diagnosis of drug use disorder (Appendix Table 2 for model coefficients and Fig. 1 for hazard ratios). The c-index was 0.75, indicating good overall discrimination. The model showed good discrimination for violent reoffending within 1 year (AUC = 0.75), 3 years (AUC = 0.76), and 5 years (AUC = 0.77) (Figs. 2 and 3 and Appendix Fig. 2). The external validation also reported good discrimination for violent reoffending (AUC = 0.77, Appendix Fig. 3) for all three follow-up periods (Barnett, Wakeling, & Howard, 2010). Other discrimination measures including sensitivity, specificity, positive predictive value (PPV), and negative predictive value (NPV) for prespecified cut-offs (5%, 10%, 15%, and 20%) are presented in Appendix Table 3.

The model showed good calibration in the large for violent reoffending predictions (Appendix Fig. 4). The expected risk/observed risk ratios ranged from 1.03 to 1.17. The Brier score was 0.040 for violent reoffending within 1 year, 0.077 for within 3 years, and 0.094 for within 5 years after the index sexual offence conviction. All Brier scores were lower than those obtained when using the mean predicted probability or using zero (Appendix Table 4). The external validation model showed similar discrimination and calibration performance (Appendix Fig. 3).

When we investigated any reoffending as a secondary outcome, included risk factors in the final model were: younger age, unemployment, low education, previous non-sexual violence, previous non-violent offending, previous imprisonment, alcohol use disorder, and drug use disorder (Appendix Table 5 for model coefficients and Appendix Fig. 5 for hazard ratios). The derivation model showed good discrimination and calibration. The c-index was 0.72. AUCs were 0.74 for 1 year, 0.75 for 3 years and 5 years, respectively (Figs. 2 and 3 and Appendix Fig. 2). The external validation reported good discrimination for any reoffending (AUC ranged from 0.76 to 0.79, Appendix Fig. 6) for all three follow-up periods. The calibration was also good (Appendix Fig. 7), with low expected risk/observed risk ratios and Brier scores (Appendix Table 4). Discrimination measures (sensitivity, specificity, PPV, and NPV) based on different cut-offs are reported in Appendix Table 6.

Finally, we tested the model for predicting sexual reoffending. The risk factors included in the final model were younger age, unemployment, previous sexual offending, previous non-violent offending, previous imprisonment, any mental disorder, and previous violent victimization (Appendix Table 7 for model coefficients and Appendix Fig. 8 for hazard ratios). The model showed moderate discrimination. The c-index was 0.67, and AUCs between 0.65 and 0.67 (Appendix Fig. 9). Measures of calibration were good (Appendix Fig. 10 and see Appendix Table 4 for expected/observed risk ratios and Brier scores), and other discrimination measures (sensitivity, specificity, PPV, and NPV) are presented in Appendix Table 8.

Three online risk tools/calculators (Oxford Risk of Recividism in Sexual Offenders or OxRIS) were created based on coefficients of the final models (see https://oxrisk.com).

## 4 Discussion

In a national cohort of all 16,231 men convicted of sex offences between 1994 and 2013 in Sweden, we examined reoffending rates, which were 15% for violent convictions, 31% for any convictions, and 4% for sexual crimes over a follow-up period of around 3 years. Using national linked registers across health, crime and sociodemographic factors, we developed risk prediction models for these three reconviction outcomes. The models performed well in terms of discrimination for violent (c-index 0.75) and any reoffending (c-index 0.72) and moderately for sexual reoffending (c-index 0.67). Calibration was good in the large for all of these models. We converted these models into online risk calculators (OxRIS tools) for the assessment of reoffending risk in sexual offenders.

In relation to other tools for sexual offending, Static-99R is a commonly used risk assessment instrument, consisting of 10 risk markers and age classified in four broad groups. Its performance is moderate for violent reoffending (as indicated by AUCs = 0.66 to 0.73, Bartosh, Garby, Lewis, & Gray, 2003; Eher, Matthes, Schilling, Haubner-MacLean, & Rettenberger, 2012; Hanson & Thornton, 1999)) and any reoffending (AUCs = 0.69 to 0.71; Bartosh et al., 2003; Eher et al., 2012)). Another tool, SORAG, which is considerably more resource-intensive, reports AUCs from 0.64 to 0.77 for violent reoffending (Barbaree, Seto, Langton, & Peacock, 2001; Bartosh et al., 2003; Eher et al., 2012; Rettenberger, Rice, Harris, & Eher, 2017). In relation to sexual recidivism, the secondary outcome, the current study reported a model with moderate predictive accuracy (AUC = 0.67). This could partly be due to the lower rate of sexual reoffending. Consistent with this, existing tools have reported low to moderate discrimination, including for Static-99/Static-99R (AUC = 0.69 [0.57–0.92]), RRASOR (0.67 [0.42–0.77]), and SORAG (0.68 [0.67–0.77]) according to a meta-review (Tully et al., 2013), and STABLE-2007, with a reported AUC of 0.62 (Brankley, Babchishin, & Hanson, 2021). The newly developed tool is different to Static-99R because it includes modifiable risk factors, such as mental and substance use disorders, enters age as a continuous factor, and was developed using a pre-determined protocol, multivariable models, and a total population-based sample of sexual offenders.

To facilitate clinical interpretation, we calculated sensitivity, specificity, PPV, NPV using different cut-off scores. Depending on different contexts and processes, clinicians and professionals in criminal justice and mental health can choose different cut-off scores to inform their decision-making. Careful consideration is needed of the differential impact of false positives or false negatives in different settings. If the consequences of being categorized as high risk are not harmful, such as additional psychosocial interventions and support, false positives can be tolerated (i.e. lower specificities are acceptable). However, it is likely that there is little societal and political tolerance of false negatives (i.e. higher sensitivities will be expected). In addition, if the goal of the risk assessment is to avoid false positives and reduce the humanitarian, legal and economic costs of punishment or incarceration, then a higher cut-off score (e.g., 20%) could be considered. If the aim is to introduce interventions to reduce recidivism, then a lower cut-off score (e.g., 5%) could be adopted. For both violent and any reoffending, the tools could achieve both high sensitivity (>80%) and specificity (>80%), depending on cut-offs, and might be useful for both identifying high risk and screening out low risk offenders. For sexual reoffending, across different cut-off scores, the tool consistently had a NPV of greater than 97%, which suggests it can be used to screen out low risk offenders, which can assist with preserving resources (e.g. by avoiding further risk assessments or expensive interventions). Alternatively, the tool could be used without cut-offs and provide a probability score that can inform decision-making as an adjunct.

The current prediction models indicate that substance use disorders and other mental disorders could possibly be a target of therapeutic interventions to reduce the risk of reoffending in men convicted of sexual crimes (Kraanen & Emmelkamp, 2011; Pickard & Fazel, 2013; Seto, 2019). For both violent and any reoffending outcomes, alcohol and drug use disorders were important risk factors and, in the model for sexual recidivism, any mental disorder was predictive of later sexual recidivism. These results suggest that treating substance use and other mental disorders may have a role in decreasing future risk, and that the tools could assist in decisions about the allocation of psychological and medical treatment and supervision, which are typically limited in most criminal justice and liaison services. In addition, the diagnosis of substance use disorders relied on patient registers. Although these registers have good diagnostic validity, it should be noted that the prevalence of substance use disorders may be underestimated. That is, men convicted of sexual crimes might have presented symptoms of, and actually met the diagnostic criteria for substance use disorders, but were not formally diagnosed.

This study has several strengths. We developed risk assessment tools with data from a large national cohort of men convicted of sex offences, allowing for more stable model performance. Furthermore, apart from discriminative validity, we also reported calibration, which is rare in this field (Fazel et al., 2022). Another strength is the tools were validated in an external sample. Previous studies are often limited by small samples and lack external validation (Helmus, 2018; Lussier, Deslauriers-Varin, & Ratel, 2010). Furthermore, compared to most currently used tools, these tools provide risk probability scores. Unlike categorizing individuals into different risk bins according to a certain cut-off score, this approach provides flexibility as clinicians and practitioners can choose the probability score and related prediction statistics to best assist decision-making according to specific contexts. In other words, what is considered a high risk will depend on the setting and resources available for supervision and management. Higher risks might be tolerated in people followed up by probation and with specified restrictions (e.g. location), but not in unsupervised persons.

Several limitations should be noted. First, we were not able to test certain factors which have been linked to sexual reoffending, such as childhood sexual abuse (Hailes, Yu, Danese, & Fazel, 2019), victim characteristics (Hanson, 1997), paraphilic and compulsive or hypersexual interest, and certain attitudes and cognitions that may be related to offending (Hanson & Morton-Bourgon, 2009). Such data are not routinely available and reliable, and require in-depth expert clinical evaluation. Second, our sample only included male sexual offenders. This is because, of the total population convicted of sexual offences, only 1% were female and risk factors may be different (Cortoni & Gannon, 2016). The low rate of female sexual offending, convictions, and recidivism will necessarily limit the development of women-specific risk assessment tools. At the same time, using tools developed from male sexual offenders will overestimate the reoffending risk of female sexual offenders (Cortoni, Hanson, & Coache, 2010) and different tools will need testing in women. One validation study of the Static-99R has reported that the tool performs poorly for women (Marshall, Miller, Cortoni, & Helmus, 2021). A further limitation is that sexual offending is largely under-reported in official records. Studies have reported substantial inconsistency between self-report survey data on sexual crimes and official registry data (DeLisi et al., 2016; Scurich & John, 2019). For example, a survey of a group of offenders with no official records of sexual abuse self-reported sexual offending against on average four victims not leading to conviction. In addition, there might be differential risk factors for detected and undetected sexual crimes due to different features in these two groups (Stephens, Klein, & Seto, 2021). Therefore, the tools reported here might need adaptation for men whose sexual crimes have not been recorded in crime registry as convicted.

Third, we developed the tools using Swedish data. Varying criminal incidence, different crime definitions, recording methods and clearance rates, and other criminal justice processes across countries will lead to different recidivism rates for sexual and other offences. Therefore, the predictive accuracy might vary and external validations need to be considered when tools are used in other countries. Fourth, although good predictive performance was reported, feasibility studies to examine the utility and acceptability of the tools are required (Helmus et al., 2021). Finally, we did not model the effect of changes over time in certain factors associated with sexual crimes (Lloyd, Hanson, Richards, & Serin, 2020; van den Berg et al., 2018), and future studies could examine this. Our findings have potential implications about the modification of certain factors, such as mental health and employment status, on improving reoffending outcomes. The results could be the basis of interventions if there are effective treatments. However, the factors in these tools are static in the sense that they are recorded at one point, and there is a need for risk monitoring and understanding links between dynamic or changeable factors on reoffending during follow-up. Nevertheless, the current findings suggest opportunities for intervention. In addition to treating substance use disorders, employment is associated with desistance from reoffending (Tripodi, Kim, & Bender, 2010; Wooditch, Tang, & Taxman, 2014).

In conclusion, using linked national data over two decades from a population-based cohort of men convicted for sexual offences, we have developed three evidence-based risk assessment tools to predict violent, any, and sexual reoffending. Methods and findings were transparently reported and followed best current practice in prediction modelling. A full range of performance measures were examined. Discriminative validity was good for both violent and any reoffending and moderate for sexual reoffending. The tools were well calibrated, particularly at lower risk levels. Based on this, we have created freely available online risk calculators (OxRIS) that provide a probability score. These tools can be used in the criminal justice system to assist decision-making at sentencing and at other points, treatment allocation including for alcohol and drug misuse and other mental disorders. Forensic mental health services may also consider their use to complement current approaches.

## Funding

SF is funded by a Wellcome Trust Senior Research Fellowship (Grant no. 202836/Z/16/Z).

## Figures and Tables

**Figure:**
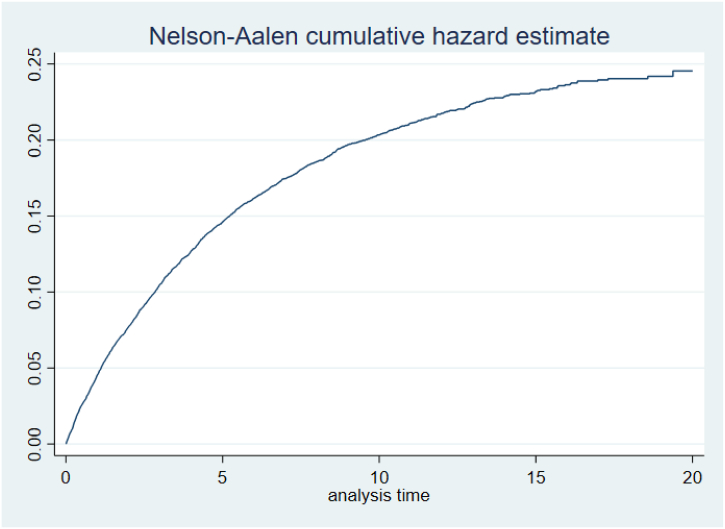
Appendix Fig. 1. Estimated probability of violent reoffending in individuals convicted of sexual offences.

**Figure:**
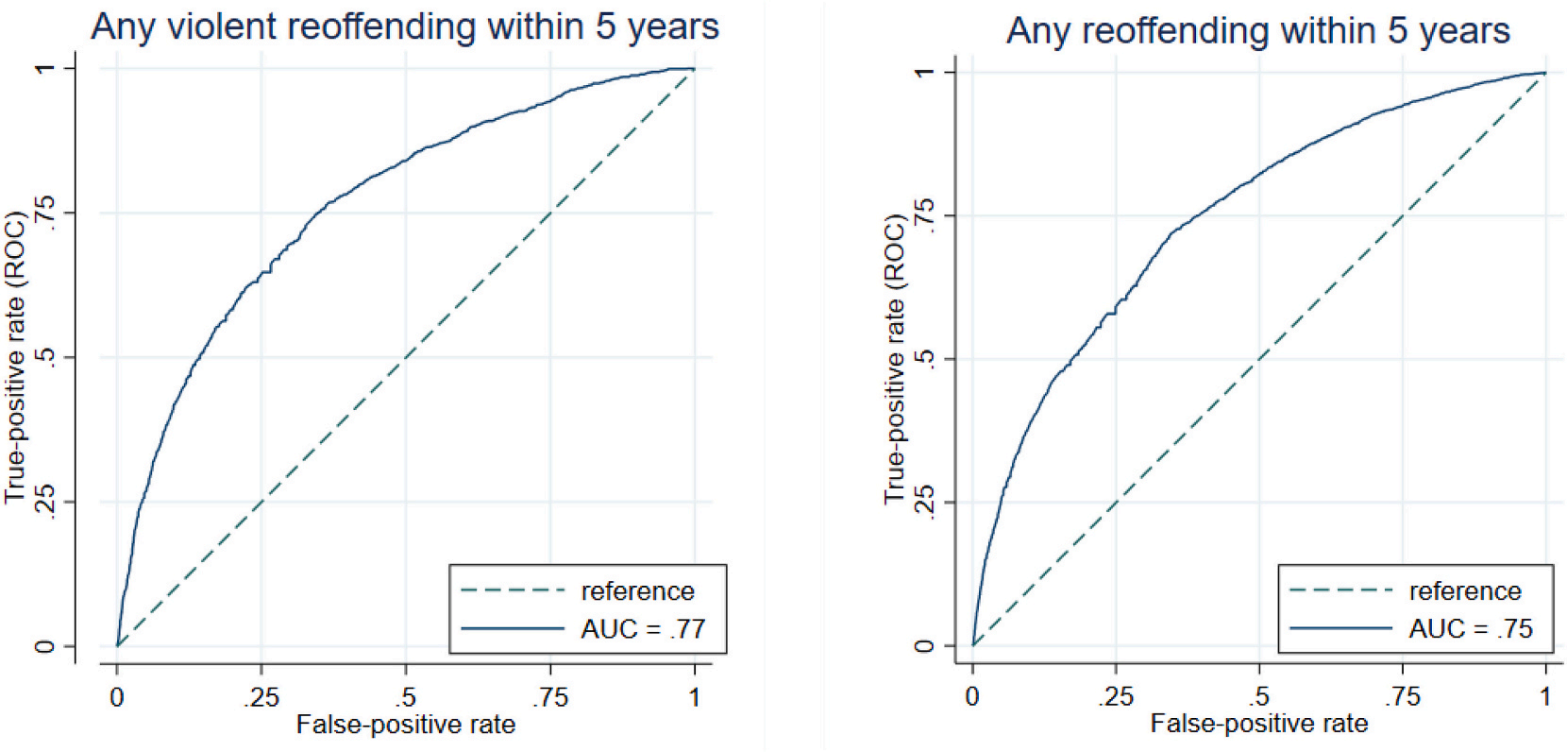
Appendix Fig. 2. Model discrimination shown by receiver operating characteristics curves for violent and any reoffending within 5 years. Note: AUC = areas under the receiver operating characteristics curves.

**Figure:**
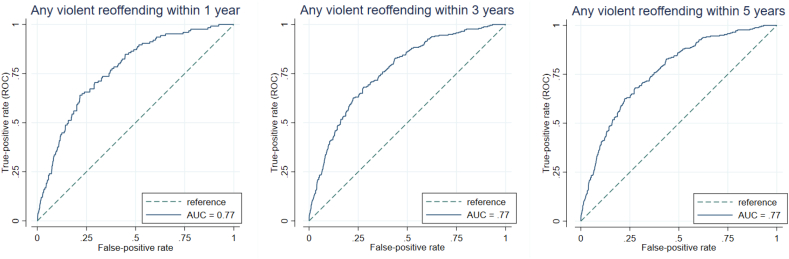
Appendix Fig. 3. Model discrimination shown by receiver operating characteristics curves for violent reoffending. Note: AUC = areas under the receiver operating characteristics curve.

**Figure:**
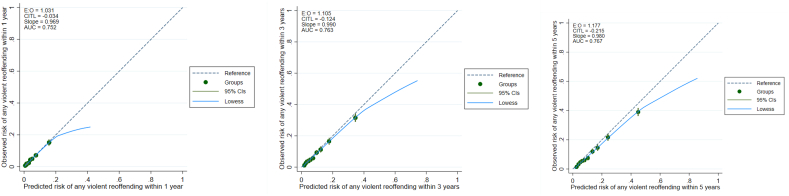
Appendix Fig. 4. Model calibration for violent reoffending.

**Figure:**
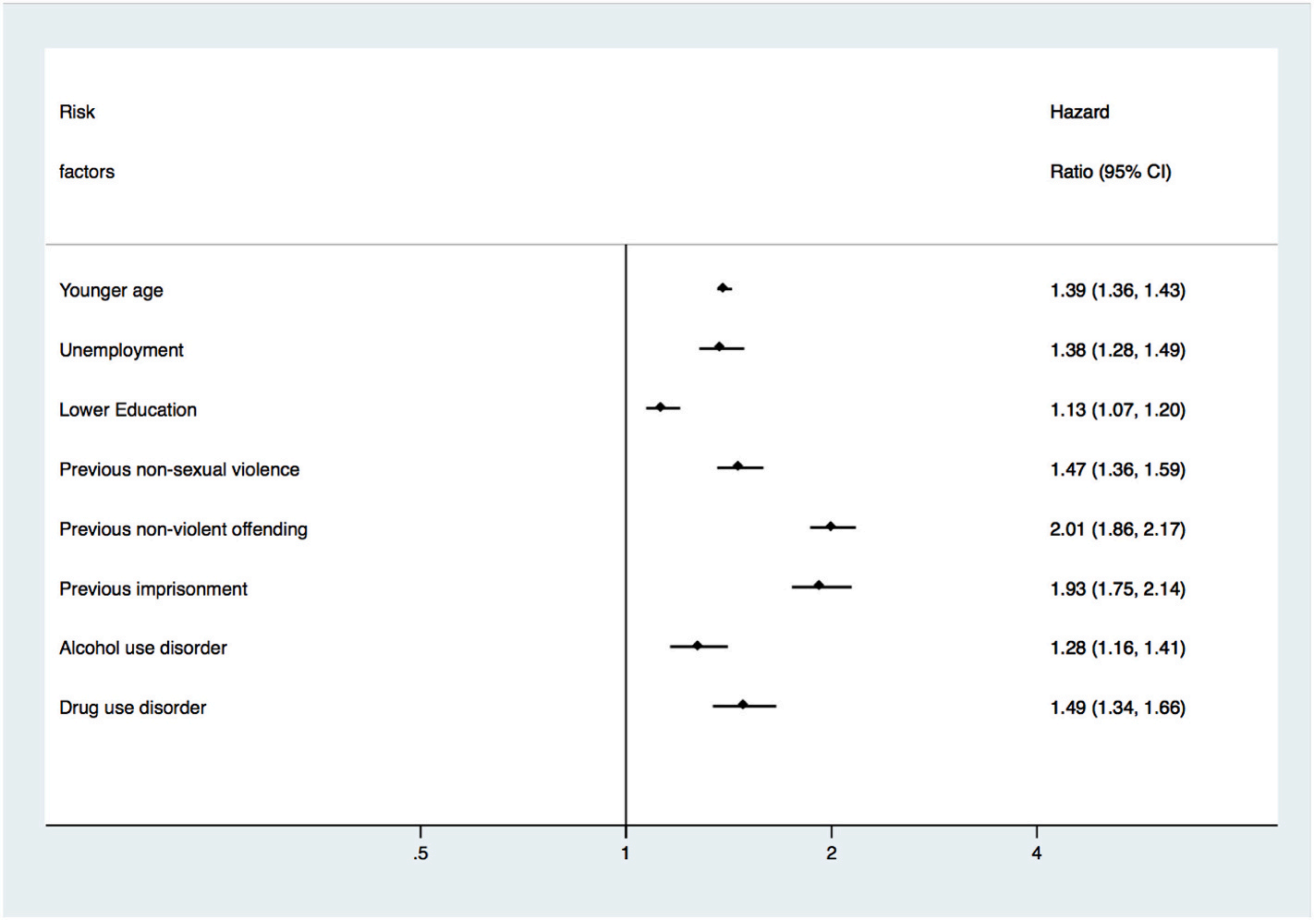
Appendix Fig. 5. Risk factors included in the final model for prediction of any reoffending and their hazard ratios. Note: Younger age refers to the effect per 10 years of age. Unemployment refers to status at time of conviction of index sexual offence.

**Figure:**
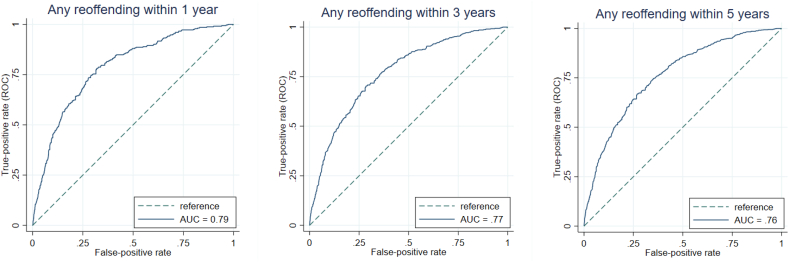
Appendix Fig. 6. Model discrimination shown by receiver operating characteristics curves for any reoffending. Note: AUC = areas under the receiver operating characteristics curve.

**Figure:**
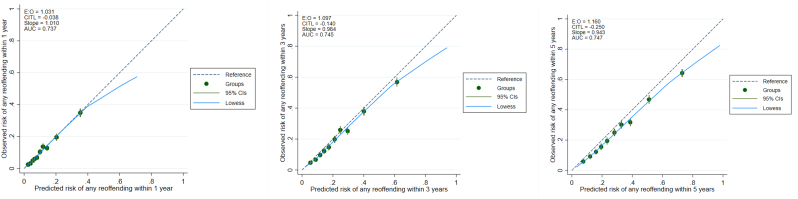
Appendix Fig. 7. Model calibration for any reoffending.

**Figure:**
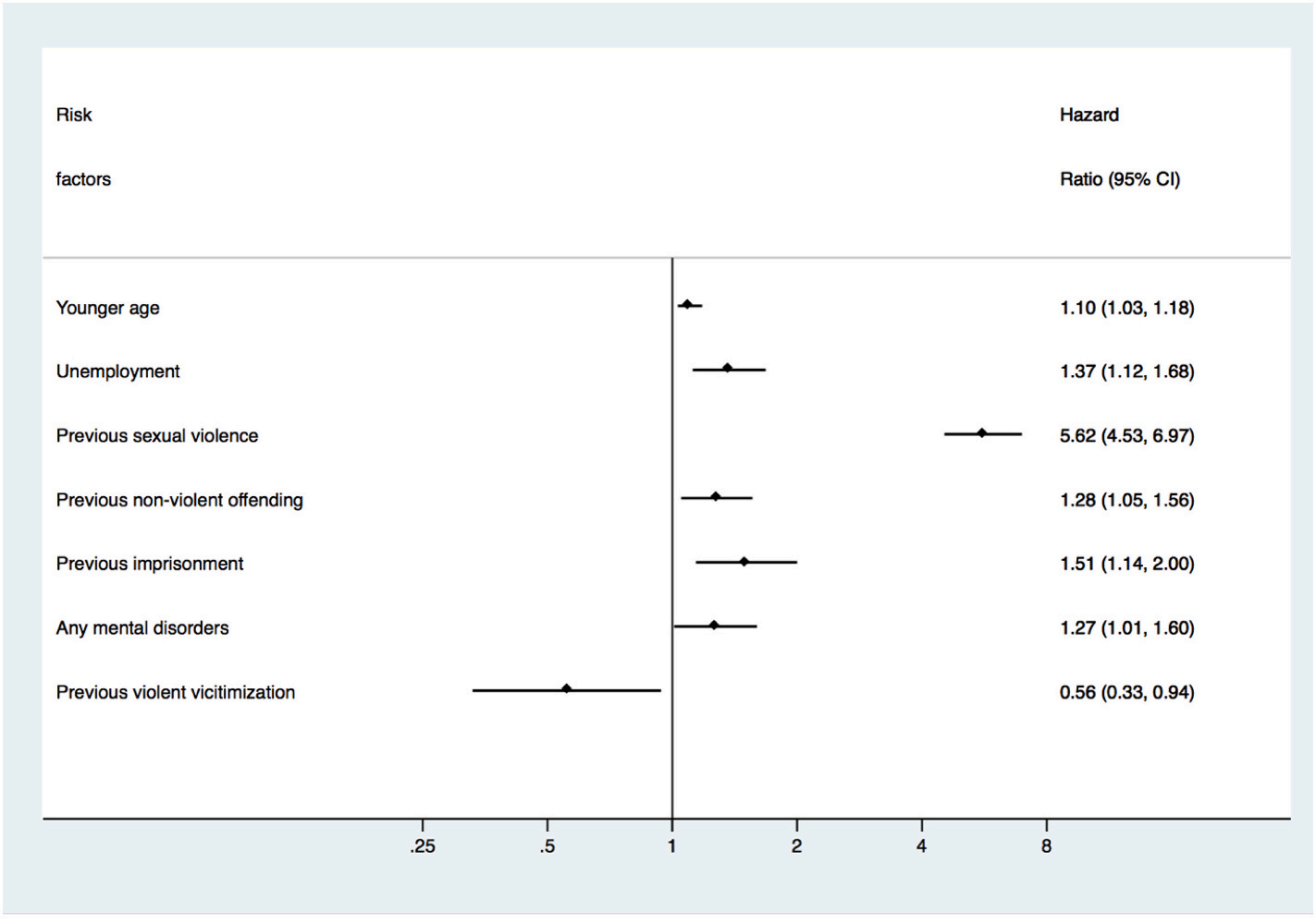
Appendix Fig. 8. Risk factors included in the final model for prediction of sexual reoffending and their hazard ratios. Note: Younger age refers to the effect per 10 years of age. Unemployment refers to status at time of conviction of index sexual offence.

**Figure:**
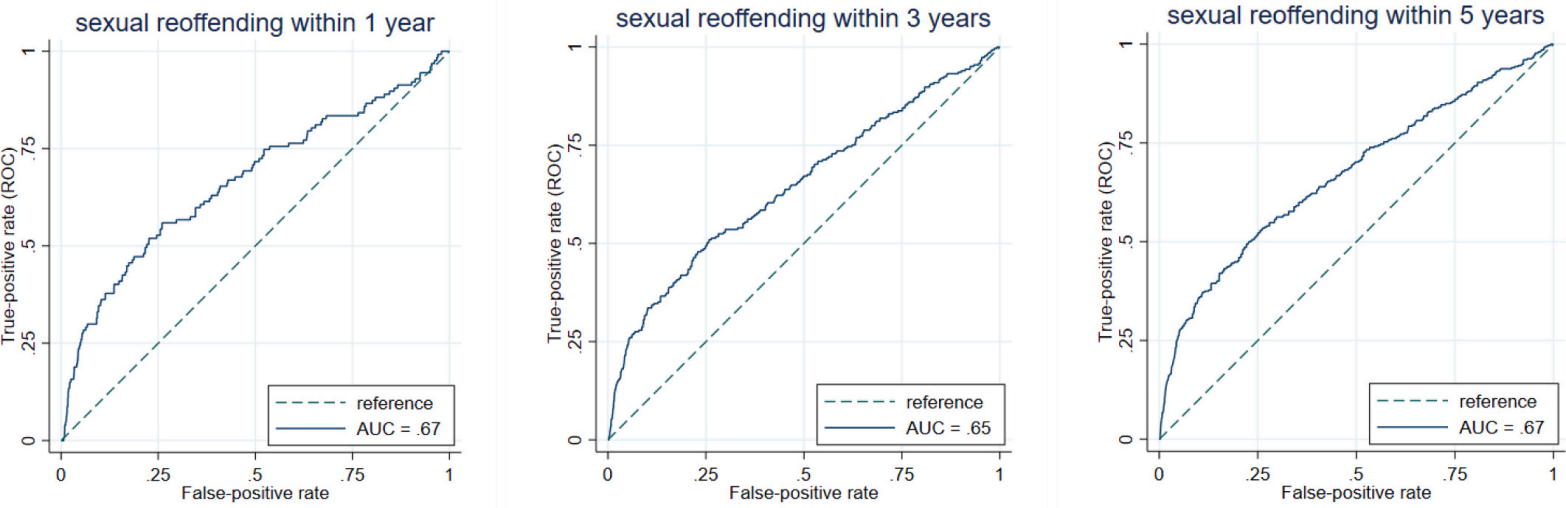
Appendix Fig. 9. Model discrimination shown by receiver operating characteristics curves for sexual reoffending.

**Figure:**
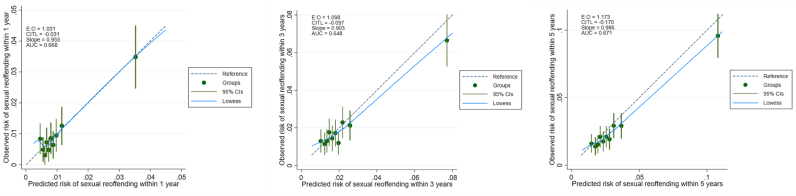
Appendix Fig. 10. Model calibration for sexual reoffending.

**Table:** Appendix Table 1 Sex offences according to the Swedish Penal Code.

Sexual offence
(aggravated) rape
(aggravated) sexual coercion
(aggravated) sexual abuse of a dependent adult
(aggravated) sexual abuse
(aggravated) sexual abuse/rape of a child (from 20,050,401 is “rape of a child”)
Sexual abuse of a child
(aggravated) sexual intercourse with a child
Child pornography offence
Sexual harassment (child or adult)
Buying of sexual service from a child
Exploitation of child for sexual posing
Sexual intercourse with offspring/ sibling
Buying of sexual service from adult
(aggravated) pimping

**Table:** Appendix Table 2 Parameters to calculate predicted risk of violent reoffending within 1, 3 and 5 years after conviction of a sexual offence.

Risk factors	Beta	LCI	UCI
Younger age	0.0341	0.0301	0.0381
Unemployment	0.4045	0.2930	0.5160
Single	0.1482	0.0001	0.2964
Education			
Post-Secondary	0	0	0
Upper-Secondary	0.1326	0.0276	0.2376
Secondary	0.3301	0.1536	0.5067
Previous sexual offending	0.4200	0.2704	0.5696
Previous non-sexual violence	0.6844	0.5728	0.7959
Previous non-violent offending	0.3760	0.2598	0.4922
Previous imprisonment	0.7045	0.5735	0.8354
Alcohol use disorder	0.3490	0.2186	0.4795
Drug use disorder	0.2983	0.1559	0.4407

Note: LCI = Lower Confidence Interval; UCI = Upper Confidence Interval.

Unemployment and single status refer to status at time of conviction of index sexual offence.

**Table:** Appendix Table 3 Sensitivity, specificity, negative predictive value, and positive predictive value by different cut-off scores for probability of violent reoffending.

	Sensitivity (95%CI)		Specificity (95%CI)		PPV (95%CI)		NPV (95%CI)	
Cut-off								
Within 1 year								
5%	61.7%	(57.4%, 65.9%)	75.8%	(75.1%, 76.6%)	9.9%	(8.9%, 11.0%)	97.7%	(97.6%, 98.2%)
10%	36.0%	(31.8%, 40.2%)	91.3%	(90.8%, 91.8%)	15.15%	(13.1%, 17.2%)	97.1%	(96.8%, 97.4%)
15%	18.8%	(15.4%, 22.2%)	96.2%	(95.9%, 96.6%)	17.66%	(14.4%, 20.9%)	96.5%	(96.2%, 96.8%)
20%	9.1%	(6.6%, 11.6%)	98.5%	(98.3%, 98.7%)	21.00%	(15.6%, 26.4%)	96.2%	(95.8%, 96.5%)
Within 3 years								
5%	87.7%	(85.8%, 89.6%)	42.1%	(41.2%, 43.1%)	13.08%	(12.3%, 13.9%)	97.2%	(96.7%, 97.7%)
10%	69.7%	(67.0%, 72.4%)	69.4%	(68.5%, 70.2%)	18.42%	(17.3%, 19.6%)	95.8%	(95.4%, 96.3%)
15%	51.2%	(48.3%, 54.2%)	84.6%	(83.9%, 85.2%)	24.79%	(23.0%, 26.6%)	94.6%	(94.1%, 95.0%)
20%	39.4%	(36.5%, 42.3%)	90.8%	(90.2%, 91.3%)	29.71%	(27.4%, 32.1%)	93.8%	(93.3%, 94.2%)
Within 5 years								
5%	94.0%	(92.7%, 95.2%)	26.2%	(25.4%, 27.0%)	14.26%	(13.6%, 15.0%)	97.1%	(96.5%, 97.7%)
10%	80.0%	(77.9%, 82.1%)	58.0%	(57.1%, 59.0%)	19.94%	(18.9%, 21.0%)	95.7%	(95.2%, 96.2%)
15%	64.1%	(61.6%, 66.6%)	75.2%	(74.4%, 76.0%)	25.26%	(23.8%, 26.7%)	94.1%	(93.6%, 94.6%)
20%	51.8%	(49.2%, 54.4%)	84.8%	(84.1%, 85.4%)	30.76%	(28.9%, 32.6%)	93.1%	(92.6%, 93.6%)

Note: Data are given in percentages. 95%CI, 95% confidence interval; PPV, positive predictive value; NPV, negative predictive value. See Table 2 for baseline rate of reoffending at different years as a reference.

**Table:** Appendix Table 4 Brier scores for outcomes at different follow-up periods.

Brier scores									
	Within1 year			Within 3 years			Within 5 years		
	Predicted probability	Mean probability	Zero probability	Predicted probability	Mean probability	Zero probability	Predicted probability	Mean probability	Zero probability
Violent reoffending	0.0400	0.0430	0.0840	0.0770	0.0930	0.0840	0.0940	0.1190	0.1050
Any reoffending	0.0930	0.1150	0.1680	0.1450	0.2130	0.1680	0.1640	0.2600	0.1940
Sexual reoffending	0.0098	0.0100	0.0205	0.0202	0.0209	0.0205	0.0263	0.0278	0.0270

**Table:** Appendix Table 5 Parameters to calculate predicted risk of any reoffending within 1, 3 and 5 years after conviction of a sexual offence.

Risk factors	Beta	LCI	UCI
Younger age	0.0332	0.0306	0.0358
Unemployment	0.3231	0.2507	0.3956
Education			
Post-Secondary	0	0	0
Upper-Secondary	0.1151	0.0454	0.1847
Secondary	0.2687	0.1426	0.3948
Previous non-sexual violence	0.3877	0.3108	0.4645
Previous non-violent offending	0.6964	0.6198	0.7730
Previous imprisonment	0.6594	0.5584	0.7605
Alcohol use disorder	0.2474	0.1525	0.3423
Drug use disorder	0.4013	0.2954	0.5073

Note: LCI = Lower Confidence Interval; UCI = Upper Confidence Interval.

Unemployment refers to status at time of conviction of index sexual offence.

**Table:** Appendix Table 6 Sensitivity, specificity, negative predictive value, and positive predictive value by different cut-off scores for probability of any reoffending.

	Sensitivity (95%CI)		Specificity (95%CI)		PPV (95%CI)		NPV (95%CI)	
Cut-off								
Within 1 year								
5%	94.8%	(93.6%, 95.9%)	24.3%	(23.5%, 25.1%)	14.0%	(13.3%, 14.6%)	97.3%	(96.7%, 97.9%)
10%	76.3%	(74.1%, 78.5%)	59.1%	(58.2%, 60.0%)	19.5%	(18.4%, 20.5%)	95.1%	(94.6%, 95.6%)
15%	51.1%	(48.5%, 53.7%)	80.7%	(80.0%, 81.5%)	25.6%	(24.0%, 27.2%)	92.7%	(92.%, 93.3%)
20%	40.8%	(38.3%, 43.4%)	88.1%	(87.5%, 88.7%)	30.0%	(28.8%, 32.9%)	92.0%	(91.5%, 92.5%)
Within 3 years								
5%	99.5%	(99.2%, 99.7%)	4.5%	(4.1%, 4.9%)	22.1%	(21.3%, 22.8%)	96.8%	(95.2%, 98.4%)
10%	95.1%	(94.2%, 95.9%)	22.6%	(21.8%, 23.5%)	25.0%	(24.2%, 25.9%)	94.4%	(93.5%, 95.3%)
15%	85.8%	(84.5%, 87.1%)	44.1%	(43.2%, 45.1%	29.5%	(28.5%, 30.5%)	92.0%	(91.2%, 92.7%)
20%	76.3%	(74.7%, 77.9%)	59.9%	(59.0%, 60.9%)	34.1%	(32.9%, 35.3%)	90.3%	(89.6%, 91.0%)
Within 5 years								
5%	99.9%	(99.8%, 100.0%)	0.7%	(0.5%, 0.8%)	26.1%	(25.4%, 26.9%)	95.4%	(90.3%, 100.0%)
10%	97.9%	(97.4%, 98.4%)	12.6%	(11.9%, 13.3%)	28.2%	(27.4%, 29.1%)	94.4%	(93.1%, 95.7%)
15%	93.4%	(92.6%, 94.3%)	27.8%	(26.9%, 28.7%)	31.3%	(30.3%, 32.2%)	92.3%	(91.%, 93.3%)
20%	85.5%	(84.3%, 86.7%)	44.9%	(43.8%, 45.7%)	35.3%	(34.2%, 36.3%)	89.8%	(89.0%, 90.7%)

Note: Data are given in percentages. 95%CI, 95% confidence interval; PPV, positive predictive value; NPV, negative predictive value. See Table 2 for baseline rate of reoffending at different years as a reference.

**Table:** Appendix Table 7 Parameters to calculate predicted risk of sexual reoffending after conviction of a sexual offence.

Risk factors	Beta	LCI	UCI
Younger age	0.0098	0.0034	0.0163
Unemployment	0.3157	0.1136	0.5179
Previous sexual violence	1.7259	1.5108	1.9410
Previous non-violent offending	0.2462	0.0447	0.4477
Previous imprisonment	0.4113	0.1280	0.6946
Any mental disorders	0.2391	0.0068	0.4715
Previous violent victimization	−0.5834	−1.1056	−0.0612

Note: LCI = Lower Confidence Interval; UCI = Upper Confidence Interval.

Unemployment refers to status at time of conviction of index sexual offence.

**Table:** Appendix Table 8 Sensitivity, specificity, negative predictive value, and positive predictive value by different cut-off scores for probability of sexual reoffending.

	Sensitivity (95%CI)		Specificity (95%CI)		PPV (95%CI)		NPV (95%CI)	
Cut-off								
Within one year								
5%	14.2%	(8.1%, 20.2%)	97.8%	(97.6%, 98.1%)	6.2%	(3.4%, 9.0%)	99.1%	(99.0%, 99.3%)
10%	0.0%	(0.0%, 0.0%)	99.94%	(99.90%, 99.99%)	0.0%	(0.0%, 0.0%)	99.0%	(98.8%, 99.2%)
Within 3 years								
5%	26.0%	(20.8%, 31.3%)	94.4%	(94.0%, 94.8%)	9.06%	(7.0%, 11.1%)	98.4%	(98.1%, 98.6%)
10%	15.1%	(10.8%, 19.4%)	97.3%	(97.1%, 97.6%)	10.78%	(7.6%, 14.0%)	98.2%	(98.0%, 98.4%)
15%	3.0%	(1.0%, 5.1%)	99.4%	(99.2%, 99.5%)	8.99%	(3.1%, 15.0%)	98.0%	(97.7%, 98.2%)
20%	0.0%	(0.0%, 0.0%)	99.9%	(99.9%, 100.0%)	0.00%	(0.0%, 0.0%)	97.9%	(97.7%, 98.2%)
Within 5 years								
5%	29.3%	(24.5%, 34.0%)	93.43%	(93.0%, 94.0%)	11.28%	(9.2%, 13.3%)	97.9%	(97.6%, 98.1%)
10%	25.3%	(20.7%, 29.8%)	95.33%	(95.0%, 96.0%)	13.38%	(10.8%, 16.0%)	97.8%	(97.6%, 98.1%)
15%	15.1%	(11.3%, 18.8%)	97.88%	(98.0%, 98.1%)	16.88%	(12.7%, 21.0%)	97.6%	(97.3%, 97.9%)
20%	6.0%	(3.5%, 8.4%)	99.29%	(99.1%, 99.4%)	19.27%	(11.9%, 26.7%)	97.4%	(97.1%, 97.7%)

Note: Data are given in percentages. 95%CI, 95% confidence interval; PPV, positive predictive value; NPV, negative predictive value. See Table 2 for baseline rate of reoffending at different years as a reference.

## Appendix A

Note: AUC = areas under the receiver operating characteristics curve.
